# Nimotuzumab plus chemotherapy and immunotherapy as first-line/neoadjuvant therapy for advanced esophageal squamous cell carcinoma

**DOI:** 10.3389/fphar.2025.1585048

**Published:** 2025-07-02

**Authors:** Qi Wang, Zhi Cui, Muhong Deng, Guoqing Zhang, Fangfang Jing, Yue Ma, Fang Pang, Quanli Han

**Affiliations:** ^1^ Department of Oncology, The First Medical Center of Chinese PLA General Hospital, Beijing, China; ^2^ Senior Department of Oncology, The Fifth Medical Center of PLA General Hospital, Beijing, China; ^3^ Department of Oncology, Medical School of Chinese PLA, Beijing, China; ^4^ Medical College, Nankai University, Tianjin, China

**Keywords:** nimotuzumab, immunotherapy, resectable, unresectable, esophageal squamous cell carcinoma

## Abstract

**Background:**

Nimotuzumab has shown promising efficacy in esophageal squamous cell carcinoma (ESCC). However, the efficacy and safety of nimotuzumab plus chemotherapy and immunotherapy as first-line/neoadjuvant therapy for patients with advanced ESCC remain unclear.

**Methods:**

We performed a real world study of patients with advanced ESCC from December 2019 to April 2024. Patients were classified into resectable and unresectable group. Dosing regimen: nimotuzumab (400 mg, Q3W) plus chemotherapy (nab-paclitaxel: 240 mg/m^2^, paclitaxel liposome: 135–175 mg/m^2^, platinum: 200–400 mg/m^2^, Q3W) and immunotherapy (PD-1/PD-L1: 200–240 mg, Q3W). Overall survival (OS) and progression-free survival (PFS) were primary endpoints, objective response rate (ORR), disease control rate (DCR), and safety were secondary endpoints.

**Results:**

Totally 55 patients were included, 15 in resectable group and 40 in unresectable group. The median follow-up was 36.70 months and 34.00 months, respectively. In resectable group, ORR was 100.0%, DCR was 100.0%, R0 resection rate was 100.00%, 1-year OS was 84.00%, 2-year OS was 74.67% with 34.46 months median OS, 1-year PFS was 84.00%, and 2-year PFS was 37.33% with 21.68 months median PFS. In unresectable group, ORR was 70.0%, DCR was 90.0%, 1-year OS was 76.70%, 2-year OS was 51.29% with 28.06 months median OS, 1-year PFS was 56.64%, and 2-year PFS was 31.15% with 14.95 months median PFS. 14 (25.5%) patients developed Grade 3–5 adverse events (AEs) not related to nimotuzumab, no serious AEs or deaths occurred.

**Conclusion:**

Our treatment combination for advanced ESCC showed a favorable survival profile, and safety was tolerable.

## Introduction

Esophageal cancer is a fatal disease and a major health challenge throughout the world (Smyth et al., 2017). In 2022, esophageal cancer ranks as the eleventh most frequent cancer and the seventh most common cause of cancer death, with approximately 511,000 new cases and 445,000 deaths globally (Bray et al., 2024). Despite improvements in treatment over past decade, the survival of esophageal cancer remains poor due to delayed symptoms and aggressiveness, with only less than 20% of patients surviving more than 5 years after diagnosis (Thrift, 2021). Due to chemotherapy resistance, the addition of targeted therapy to chemotherapy has a 5-year survival rate of 15%–25% (Huang and Fu, 2019). Esophageal squamous cell carcinoma (ESCC) arises from the lining of the esophageal squamous epithelium (Yang et al., 2020), accounts for 85% of esophageal cancer cases worldwide (Zhou et al., 2023), and primarily occurs in East Asia (He et al., 2021). After treatment, ESCC is still prone to recurrence, metastasis and drug resistance, leading to poor prognosis.

Currently, chemotherapy is the primary treatment for esophageal cancer, which has the characteristics of preventing tumor growth and inhibiting distant metastasis, but it can cause dose-limiting toxicity (He et al., 2021). Platinum/fluoropyrimidine-based chemotherapy with or without immune checkpoint inhibitors is the first-line treatment standard for esophageal cancer (Puhr et al., 2023). For patients with ESCC, the addition of anti-programmed cell death protein 1 (PD-1) antibody to first-line chemotherapy reduced the relative risk for death by about 25%–40% (Shah et al., 2023). In recent years, targeted therapy for ESCC has emerged. Epidermal growth factor receptor (EGFR), an essential therapeutic target in the anticancer pathway, is associated with tumor cell proliferation, differentiation, apoptosis, and angiogenesis (Ayati et al., 2020; Zhang et al., 2018). The overexpression of EGFR has been confirmed to be related to a variety of solid human tumors, including esophageal cancer (Wang et al., 2023a). Nimotuzumab, also known as h-R3, is an anti-EGFR humanized monoclonal antibody with anti-tumor effects (Ramakrishnan et al., 2009; Tundidor et al., 2014). A phase I study suggested that nimotuzumab plus chemoradiotherapy was tolerable and effective for patients with esophageal cancer (Kato et al., 2018). Another phase Ⅲ clinical trial exhibited that nimotuzumab combined with chemoradiotherapy in the treatment of locally advanced ESCC can improve short-term efficacy and offer tolerable toxicity (Meng et al., 2023).

Here, we conducted a real world study to explore the survival and safety of nimotuzumab plus chemotherapy and immunotherapy as first-line/neoadjuvant therapy for patients with advanced ESCC.

## Materials and methods

### Patient selection

Between December 2019 and April 2024, the clinical data of patients with advanced ESCC who received nimotuzumab plus chemotherapy and immunotherapy as first-line/neoadjuvant therapy were retrospectively analyzed. According to NCCN Guidelines (Version 2), patients were divided into two groups: resectable and unresectable group. The main inclusion criteria: a) ≥ 18 years of old; b) histologically and pathologically diagnosed as naïve ESCC with clinical stage Ic-IVb; c) Eastern Cooperative Oncology Group performance status (ECOG PS) score of 0–2; d) the follow-up information was complete.

This study was approved by the institutional review board of the First Medical Center of Chinese PLA General Hospital. The research was carried out in accordance by the Helsinki Declaration and Good Clinical Practice Guidelines.

### Treatment

All patients received nimotuzumab plus chemotherapy and immunotherapy. Nimotuzumab was administered 400 mg, once every 3 weeks. The chemotherapy scheme was nab-paclitaxel (240 mg/m^2^), paclitaxel liposome (135–175 mg/m^2^), and platinum (200–400 mg/m^2^), once every 3 weeks. Physician was allowed for dose modification based on patient ECOG PS. Immunotherapy schemes were PD-1 and programmed cell death 1 ligand 1 (PD-L1), 200–240 mg, once every 3 weeks, including camrelizumab, nivolumab, pembrolizumab, toripalimab, tislelizumab, and sintilimab.

### Outcome evaluation

The primary endpoints included overall survival (OS) and progression‐free survival (PFS), and the secondary endpoints were objective response rate (ORR), disease control rate (DCR), and safety. OS was defined as the time from the start of treatment to death from any cause, and PFS was defined as the time from the start of treatment to disease progression or death from any cause, whichever occurred first. The treatment response was evaluated using The Response Evaluation Criteria in Solid Tumors (RECIST, version 1.1) (Schwartz et al., 2016), and classified into complete response (CR), partial response (PR), stable disease (SD), and progressive disease (PD). Adverse events (AEs) were estimated according to the Common Terminology Criteria for Adverse Events (CTCAE, version 5.0) (Freites-Martinez et al., 2021).

### Statistical analysis

All the statistical analyses were performed by SAS software (version 9.4). Kaplan-Meier method was performed for survival analysis, with the 95% confidence interval (CI) calculated. The Kaplan-Meier curve was conducted by GraphPad Prism 9 software. Logistic regression analysis was applied to evaluate the response. Medians were used to describe continuous variables, and percentages were used to describe categorical variables. Safety data was evaluated based on the descriptive analysis.

## Results

### Patient characteristics

**FIGURE 1:**
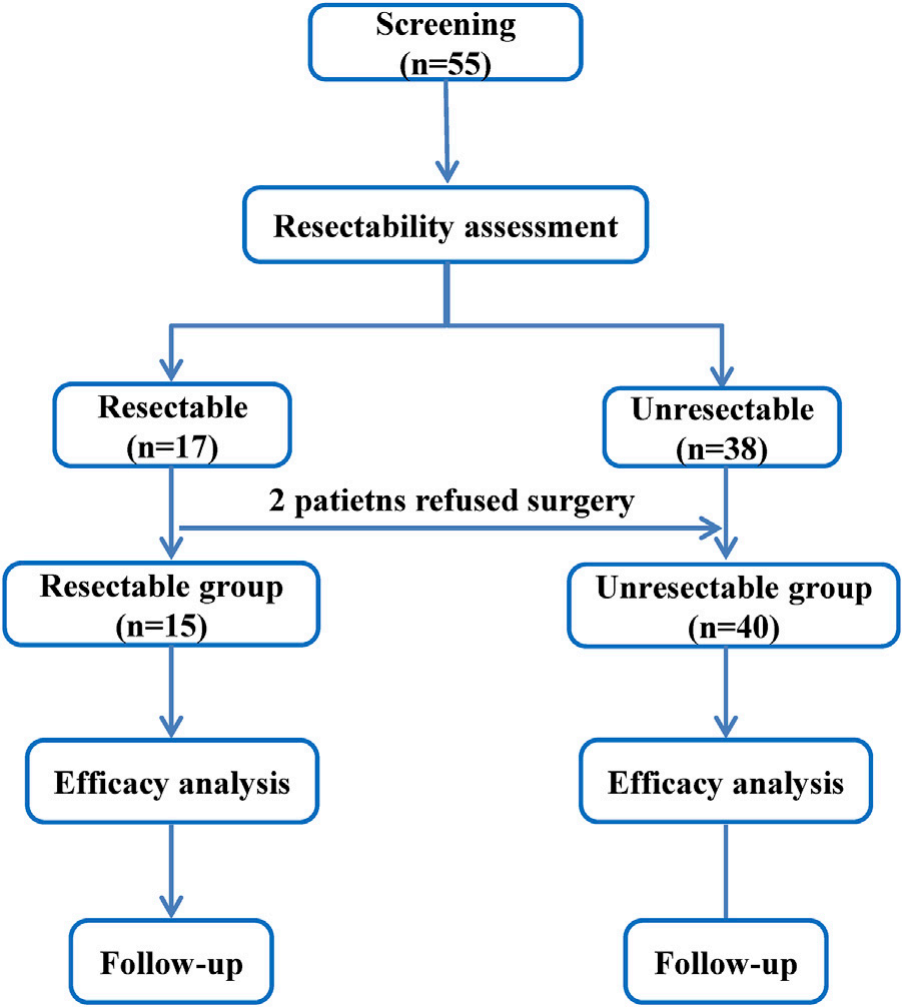
The flowchart.

A total of 55 patients with advanced ESCC were screened, according to NCCN Guidelines Version 2, 15 patients in resectable group and 40 patients in unresectable group (Figure 1). By the last follow-up on Nov 11 2024, the median follow-up was 36.70 months (95%CI: 7.89, 46.95) and 34.00 months (95%CI: 23.56, 38.93), respectively. Table 1 reported the patient baseline characteristics and demographics. The median age was 54.0 years (range: 49–73) and 60.0 years (44–80) in two groups, respectively. 1 (6.67%) patient received radiotherapy in resectable group after surgery and 23 (57.50%) patients received radiotherapy in unresectable group. In resectable group, the proportion of patients treated with nimotuzumab for 1–3 cycles and 4–6 cycles was 40.00% and 60.00%, respectively, and 25.00% and 75.00% in unresectable group.

**TABLE 1:** Baseline patient demographics and clinical characteristics.

Characteristics		Resectable group	Unresectable group
		N = 15	N = 40
Gender, n (%)			
	Male	13 (86.67%)	39 (97.50%)
	Female	2 (13.33%)	1 (2.50%)
Age	Median (range)	54.0 (49–73)	60.0 (44–80)
KPS, n (%)			
	70–80	0 (0.00%)	3 (7.50%)
	90–100	15 (100.0%)	37 (92.50%)
ECOG PS, n (%)			
	0	10 (66.67%)	21 (52.50%)
	1	5 (33.33%)	18 (45.00%)
	2	0 (0.00%)	1 (2.50%)
Smoking history, n (%)			
	No	4 (26.67%)	14 (35.00%)
	Yes	11 (73.33%)	26 (65.00%)
Alcohol history, n (%)			
	No	6 (40.00%)	11 (27.50%)
	Yes	9 (60.00%)	29 (72.50%)
T stage, n (%)			
	T1-2	12 (80.00%)	30 (81.08%)
	T3-4	3 (20.00%)	7 (18.92%)
N stage, n (%)			
	N0-1	11 (73.33%)	22 (57.89%)
	N2-3	4 (26.67%)	16 (42.11%)
M stage, n (%)			
	M0	15 (100.00%)	23 (57.50%)
	M1	0 (0.00%)	17 (42.50%)
Differentiation, n (%)			
	Low	1 (16.67%)	4 (26.67%)
	High	0 (0.00%)	2 (13.33%)
	Middle	5 (83.33%)	9 (60.00%)
Radiotherapy, n (%)			
	No	14 (93.33%)	17 (42.50%)
	Yes	1 (6.67%)	23 (57.50%)
Nimotuzumab cycle, n (%)			
	1–3	6 (40.00%)	10 (25.00%)
	4–6	9 (60.00%)	20 (75.00%)

### Response

**FIGURE 2:**
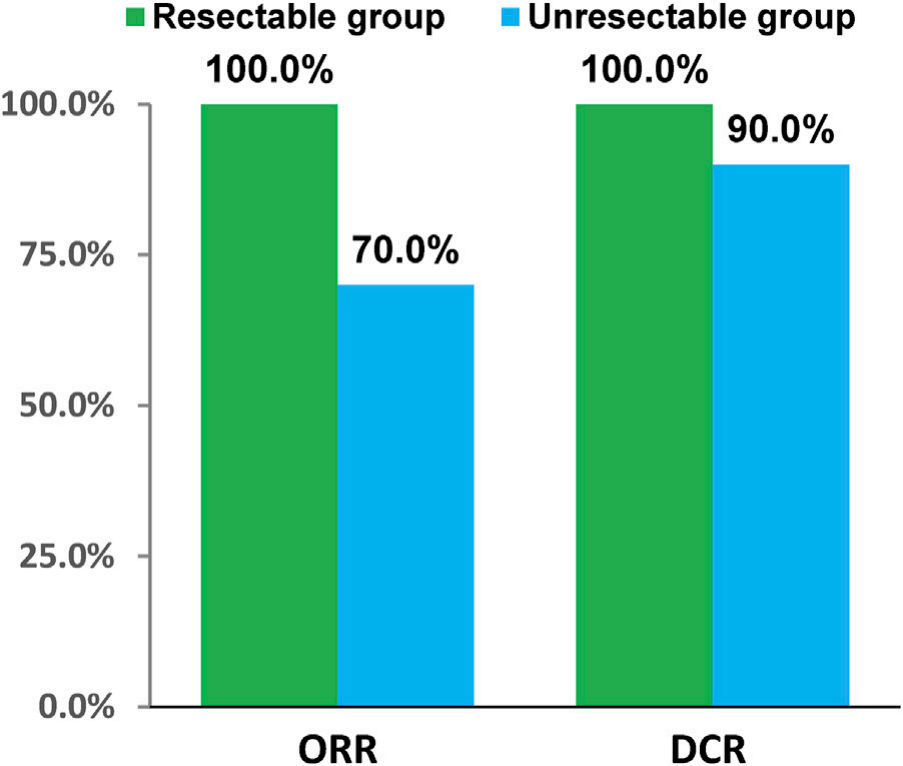
The short-term efficacy.

**FIGURE 3:**
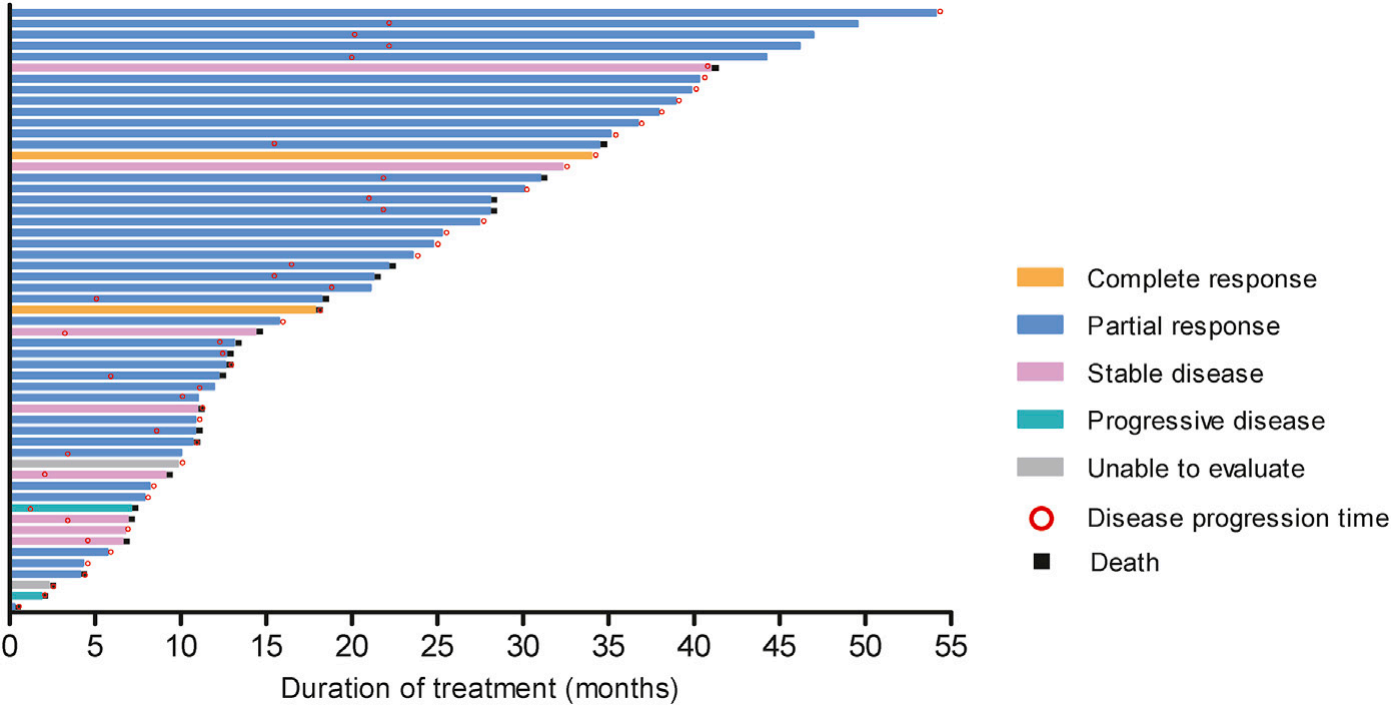
The tumor response and treatment duration.

In resectable group, CR had 1 (6.67%) patient and PR had 14 (93.33%) patients. In unresectable group, the number of CR, PR, SD and PD patients were 2 (5.00%), 26 (65.00%), 8 (20.00%), and 2 (5.00%), respectively. Short-term efficacy showed that ORR was 100.0% (95%CI: 78.20%, 100.00%), DCR was 100.0% (95%CI: 78.20%, 100.00%), R0 resection rate was 100.00%, and pathological complete response (pCR) rate was 16.67% in resectable group. Meanwhile ORR was 70.0% (95%CI: 53.47%, 83.44%) and DCR was 90.0% (95%CI: 76.34%, 97.21%) in unresectable group (Figure 2). The tumor response and treatment duration of each patient were displayed in Figure 3.

### Survival profile

**FIGURE 4:**
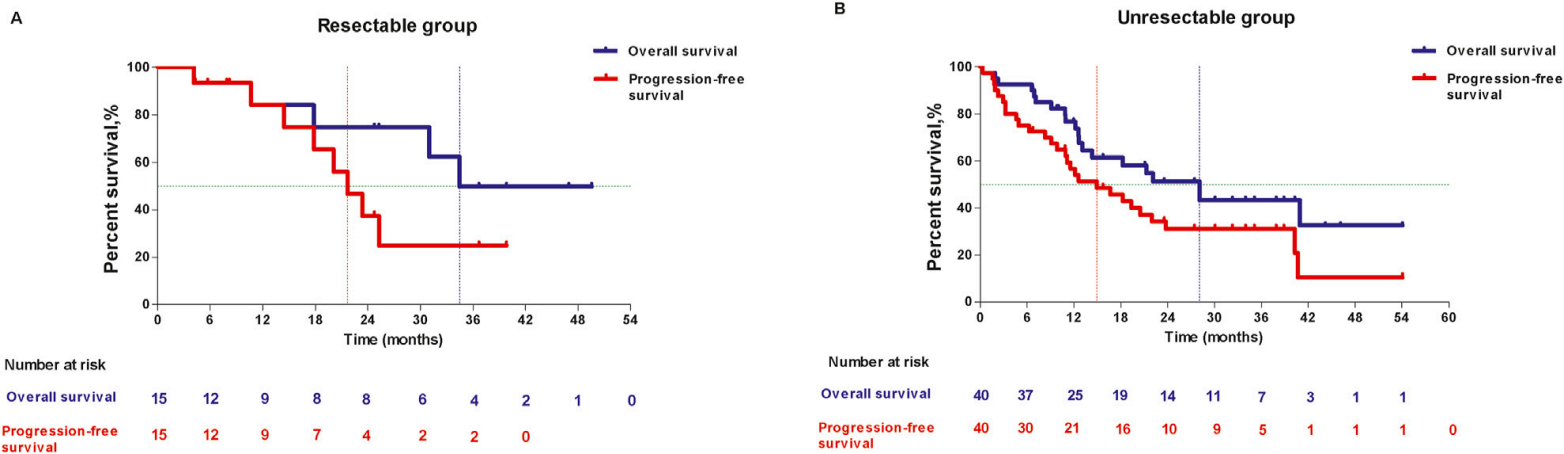
Kaplan-Meier curves of overall survival and progression-free survival in resectable group **(A)** and unresectable group **(B)**.

In resectable group, the 1-year OS and 2-year OS was 84.00% (95%CI: 48.7, 95.9) and 74.67% (95%CI: 39.5, 91.2), respectively, the median OS was 34.46 months (95%CI: 10.68, NE). 1-year PFS were 84.00% (95%CI: 48.7, 95.9) and 2-year PFS was 37.33% (95%CI: 11.6, 63.7), the median PFS was 21.68 months (95%CI: 10.68, NE). In unresectable group, 1-year OS and 2-year OS were 76.70% (95%CI: 59.9, 87.2) and 51.29% (95%CI: 33.4, 66.6), respectively, and median OS was 28.06 months (95%CI: 12.65, NE). 1-year PFS was 56.64% (95%CI: 39.8, 70.4) and 2-year PFS was 31.15% (95%CI: 17.1, 46.3), the median PFS was 14.95 months (95%CI: 9.82, 21.98). (Figure 4).

### Toxicity

During the treatment period, no serious adverse events (AEs) or deaths were observed. The most common AEs were myelosuppression (30/54.5%), nausea (33/60.0%), emesis (11/20.0%), inappetence (12/21.8%), hemoglobin decreased (10/8.2%), neutropenia (14/25.5%), leukopenia (24/43.6%), and thrombocytopenia (17/30.9%). There were 14 (25.5%) patients who developed Grade 3–5 AEs not related to nimotuzumab, of which 5 patients in the resectable group, and 9 patients in the unresectable group (Table 2).

**TABLE 2:** The most common adverse events.

Adverse events	Resectable group	Unresectable group
	N = 15	N = 40
Grade 1–2	10 (66.7%)	31 (77.5%)
Grade 3–5	5 (33.3%)	9 (22.5%)
Myelosuppression	8 (53.3%)	22 (55.0%)
Nausea	6 (40.0%)	27 (67.5%)
Emesis	2 (13.3%)	9 (22.5%)
Inappetence	2 (13.3%)	10 (25.0%)
Hemoglobin decreased	3 (20.0%)	7 (17.5%)
Rash	0 (0.0%)	6 (15.0%)
Neutropenia	3 (20.0%)	11 (27.5%)
Leukopenia	5 (33.3%)	19 (47.5%)
Thrombocytopenia	4 (26.7%)	13 (32.5%)
Hemoptysis	0 (0.0%)	2 (5.0%)
Liver injury	1 (6.7%)	3 (7.5%)
Pneumonia	0 (0.0%)	2 (5.0%)
Immune hepatitis	1 (6.7%)	0 (0.0%)
Fatigue	1 (6.7%)	0 (0.0%)

## Discussion

In this real world study, the clinical efficacy and safety of nimotuzumab plus chemotherapy and immunotherapy as first-line/neoadjuvant therapy was evaluated in advanced ESCC. We observed favorable survival outcomes, and the toxicity profile is acceptable and tolerable.

As a prevalent malignancy of the digestive system, esophageal cancer is often diagnosed with advanced stages and metastases, leading to a poor prognosis (Wang et al., 2023b). Patients with esophageal cancer often have co-morbidities, which limits the choice of treatment methods. Chemotherapy plus PD-1 has been the standard first-line therapy for this population (Shah, 2015).

A real world study showed that PD-1 plus chemotherapy had favorable efficacy in operable locally advanced ESCC, with the ORR was 70.6%, DCR was 100%, R0 resection rate was 92.3%, and the pCR rate was 15.4% (Wu et al., 2021). In a phase-II study of chemotherapy plus PD-1 for resectable ESCC, patients were given toripalimab plus paclitaxel and cis-platinum prior to surgery for two cycles, the results exhibited that the R0 resection rate was 100%, and 33.3% patients achieved pCR (Liu et al., 2021). According to the HCHTOG1909 study, toripalimab plus chemotherapy improved the survival profile for resectable esophageal cancer, with the 1-year event-free survival was 77.9%, and 1-year OS rates were 94.1% (Zheng et al., 2024). Meanwhile, a single-arm phase 2 study estimated the efficacy of toripalimab combined with nab-paclitaxel and S-1 for patients with resectable ESCC, indicted that pCR was 29.09%, R0 resection rate was 98.21%, major pCR was 49.09%, DCR was 96.67%, and the 1-year OS, PFS and DFS were 86.7%, 89.7% and 86.2%, respectively (Zhang et al., 2023).

Moreover, nivolumab plus chemotherapy for unresectable advanced and metastatic ESCC indicated the improved ORR (72%) and median OS (609 days) (Kao et al., 2023). Xi et al. found that toripalimab plus chemoradiotherapy for unresectable locally advanced ESCC displayed encouraging efficacy of clinical complete response was 60.7% (Xi et al., 2021). A retrospective analysis revealed that chemotherapy plus PD-1 for locally advanced unresectable ESCC can receive better conversion surgery rate (81.3% vs. 66.7%), ORR (73.5% vs. 48.9%), and 2-year event-free survival (76.4% vs. 42.4%) compared with chemotherapy alone (Xu et al., 2024). In the ATTRACTION-1 trial, PD-1 inhibitor nivolumab combined with chemotherapy for unresectable esophageal cancer had a trend toward longer survival with the 5-year OS was 6.3% and 5-year PFS was 6.8% (Ikeda et al., 2024).

Recently, the addition of targeted therapy regimens to chemotherapy improves the survival of patients with advanced esophageal cancer (Zhang et al., 2016). EGFR is emerging as an effective target for the treatment of esophageal cancer, and its overexpression may indicate poor survival (Hong et al., 2013). Nimotuzumab, an EGFR-targeted antibody, presented anti-tumor efficacy in esophageal cancer (Chen et al., 2024). Nimotuzumab has been found to reduce PD-L1 expression during tumor progression or chemotherapy and to increase tumor sensitivity to docetaxel and atezolizumab (Hu et al., 2024). The NXCEL1311 study suggested that nimotuzumab plus chemoradiotherapy in unresectable locally advanced ESCC had increased CRR (32.5%) and ORR (93.8%), with a good safety profile (Meng et al., 2023). Wang et al. explored the efficacy of camrelizumab combined with nimotuzumab in the second-line therapy of advanced ESCC, observed a trend benefit for ORR (36%) and favorable median OS (12.62 months) (Wang et al., 2024). Therefore, the above results provide a basis for nimotuzumab in the first-line study of advanced esophageal cancer.

In the resectable group of our study, ORR was 100.0%, DCR was 100.0%, and pCR was 16.67%. However, unlike the prospective studies, patient’s compliance in retrospective studies is limited by various realistic conditions. Patients in the resectable group were planned to receive two cycles of nimotuzumab prior to surgery, and sequentially four cycles after surgery. But in fact, only 20% (3/15) patients received complete treatment not due to toxicity and progression. Although the pCR was not satisfied in our study, it was reported that pCR does not necessarily transform into long-term survival (Tang et al., 2023). Our results of long-term survival displayed that 1-year OS and 2-year OS were 84.00% and 74.67%, respectively, 1-year PFS was 84.00% and 2-year PFS was 37.33%. Although the long-term survival was not superior to that in previous studies, most patients received only a few cycles of nimotuzumab plus PD-1 and chemotherapy (1–3 cycles: 40%, 4–5 cycles: 40%, and six cycles: 20%), but they could achieve similar long-term survival results with regular six cycles of chemotherapy plus PD-1 in the prospective randomized study. The ORR (100%), DCR (100%), and R0 resection rate (100%) in this study were also favorable.

In the unresectable group, our survival results were significantly better than the previous study. The multi-center phase 3 JUPITER-06 trial demonstrated that toripalimab in combination with chemotherapy in treatment-naïve advanced ESCC had the median OS of 17 months, and median PFS of 7 months. The 1-year OS was 66.0% and 1-year PFS was 27.8% (Wang et al., 2022). Additionally, the EGFR-targeted drug HLX07 plus PD-1 and chemotherapy in the treatment of unresectable or metastatic locally advanced ESCC indicated the antitumor activity, with the ORR was 55.2% (Huang et al., 2023). Our results for OS (1-year OS: 76.70%, 2-year OS: 51.29%, median OS: 28.06 months), PFS (1-year PFS: 56.64%, 2-year PFS: 31.15%, median PFS: 14.95 months), and ORR (70.0%) showed survival benefit than those for PD-1/EGFR-targeted drug plus chemotherapy. It is highly likely to be an effective choice for the first-line treatment of advanced esophageal cancer.

Regarding the safety analysis, nimotuzumab suggested manageable and tolerable toxicity, with no unpredictable toxicities or death occurring. A previous study found that nimotuzumab was well tolerated in patients with different epithelial tumors (Mazorra et al., 2018). In particular, nimotuzumab has a low incidence of severe AEs, with no serious skin toxicity has been reported (Allan, 2005). Other anti-EGFR agents usually have severe skin toxicities, which have been confirmed as a surrogate marker of antitumor effect (Lenz, 2006; Saif and Kim, 2007). The low toxicity of nimotuzumab may be because of its intermediate affinity, or a different mechanism of inhibition (Talaver et al., 2009). To our knowledge, immunotherapy has unique toxicities that may lead to different organ-specific inflammatory side effects (Kennedy and Salama, 2020), nevertheless, our treatment combination reduced AEs and improved patient tolerability. In our study, the main common AEs were myelosuppression, nausea, emesis, inappetence, hemoglobin decreased, neutropenia, leukopenia, and thrombocytopenia. According to the existing literature (Meng et al., 2023; Yang et al., 2021), we observed similar AEs in our study. As a result, the safety of nimotuzumab in our finding was generally consistent with its known profiles.

As an exploration study, the results showed a direction of nimotuzumab combined with immunotherapy and chemotherapy as first-line therapy in ESCC for clinical reference. In a phase 2 study, a chimeric EGFR mAb in combination with serplulimab and chemotherapy as a first-line therapy for advanced ESCC showed encouraging efficacy and manageable safety (Liu et al., 2024). Nimutuzumab as a humanized EGFR mAb with less toxicity showed a trend benefit for ORR and OS in the second-line treatment of advanced ESCC (Wang et al., 2024). Our study was the first exploration in the combination regimen as first-line treatment of advanced ESCC, maybe with great significance for treatment option in this population in the future. Furthermore, a large prospective trial of this combination regimen as first-line/neoadjuvant therapy in advanced ESCC is needed to verify the efficacy in the future.

There have several limitations in our study. As a retrospective study, the sample size is small, and the treatment compliance is not good, thus a randomized prospective study to confirm this regimen’s efficacy is needed to be conducted. Nimotuzumab has been launched to the market for many years in the worldwide, but drug resistant has not reported yet. Even so, the longtime use of nimotuzumab, that could lead tumors to develop resistance against EGFR pathway over time through mutations or activation of alternative pathways should be concerned.

Nimotuzumab plus chemotherapy and immunotherapy may have a prolonged survival profile and good therapeutic response in the first-line/neoadjuvant treatment of patients with advanced ESCC, and the safety is acceptable and manageable. Our combination regimen might offer a basis for further exploration in this population.

## Data Availability

The raw data supporting the conclusions of this article will be made available by the authors, without undue reservation.
